# Medically Complex Pregnancies and Early Breastfeeding Behaviors: A Retrospective Analysis

**DOI:** 10.1371/journal.pone.0104820

**Published:** 2014-08-13

**Authors:** Katy B. Kozhimannil, Judy Jou, Laura B. Attanasio, Lauren K. Joarnt, Patricia McGovern

**Affiliations:** 1 Division of Health Policy and Management, University of Minnesota School of Public Health, Minneapolis, Minnesota, United States of America; 2 Harvard University, Cambridge, Massachusetts, United States of America; 3 Division of Environmental Health Sciences, University of Minnesota School of Public Health, Minneapolis, Minnesota, United States of America; Oslo University Hospital, Ullevål, Norway

## Abstract

**Background:**

Breastfeeding is beneficial for women and infants, and medical contraindications are rare. Prenatal and labor-related complications may hinder breastfeeding, but supportive hospital practices may encourage women who intend to breastfeed. We measured the relationship between having a complex pregnancy (entering pregnancy with hypertension, diabetes, or obesity) and early infant feeding, accounting for breastfeeding intentions and supportive hospital practices.

**Methods:**

We performed a retrospective analysis of data from a nationally-representative survey of women who gave birth in 2011–2012 in a US hospital (N = 2400). We used logistic regression to examine the relationship between pregnancy complexity and breastfeeding. Self-reported prepregnancy diabetes or hypertension, gestational diabetes, or obesity indicated a complex pregnancy. The outcome was feeding status 1 week postpartum; *any* breastfeeding was evaluated among women intending to breastfeed (N = 1990), and *exclusive* breastfeeding among women who intended to exclusively breastfeed (N = 1418). We also tested whether breastfeeding intentions or supportive hospital practices mediated the relationship between pregnancy complexity and infant feeding status.

**Results:**

More than 33% of women had a complex pregnancy; these women had 30% lower odds of intending to breastfeed (AOR = 0.71; 95% CI, 0.52–0.98). Rates of intention to exclusively breastfeed were similar for women with and without complex pregnancies. Women who intended to breastfeed had similar rates of any breastfeeding 1 week postpartum regardless of pregnancy complexity, but complexity was associated with >30% lower odds of exclusive breastfeeding 1 week among women who intended to exclusively breastfeed (AOR = 0.68; 95% CI, 0.47–0.98). Supportive hospital practices were strongly associated with higher odds of any or exclusive breastfeeding 1 week postpartum (AOR = 4.03; 95% CI, 1.81–8.94; and AOR = 2.68; 95% CI, 1.70–4.23, respectively).

**Conclusions:**

Improving clinical and hospital support for women with complex pregnancies may increase breastfeeding rates and the benefits of breastfeeding for women and infants.

## Introduction

Breastfeeding has many advantages to infants [1]. In 2010, approximately 77% of US infants were breastfed at least once, a substantial increase from 64% in 1998 [2], [3]. Despite this progress, breastfeeding continues to fall short of national goals for duration and exclusivity set in initiatives such as Healthy People 2020 [2], [4]. One possible reason for failure to consistently meet these goals is the rise in complications women face as they enter pregnancy, including diabetes, obesity, and hypertension. Breastfeeding initiation rates are lower and breastfeeding duration is generally shorter among women with these conditions [5]–[8]. Six percent of births are complicated by diabetes [9], 3%–5% of pregnant women have hypertensive disorders [10]–[12], and 19%–39% of are obese when they become pregnant [13]. Clinical management of these conditions and associated complications may necessitate greater intrapartum or neonatal intervention, which could affect care for the woman or infant in the immediate postpartum period, including breastfeeding [14]–[19].

The decision to breastfeed is highly personal and affected by many factors, including anticipated barriers to or support for breastfeeding, hospital practices, medical issues occurring either before or during pregnancy, and complications during labor and delivery [1], [20]–[26]. One program that has been successful in encouraging breastfeeding is the Baby-Friendly Hospital Initiative (BFHI), a global program to encourage and recognize hospitals that have policies to provide evidence-based care to support infant feeding and mother-baby bonding [1], [20], [24], [25], [27]. The program, for example, instructs mothers on breastfeeding, allows babies to spend the first hour after birth in their mothers arms; provides newborns no food or drink other than breast milk, unless medically indicated; practices “rooming in” by allowing mothers and infants to remain together 24 hours per day; gives no pacifiers or artificial nipples to breastfeeding infants; and refer mothers to breastfeeding support groups on discharge from the hospital or clinic. Greater adoption of these practices is also a focus of Healthy People 2020 [28]. Yet despite the success of these measures, fewer than 7% of U.S. births currently occur in facilities with an official BFHI designation [28]. This study examines the relationship between entering pregnancy with complicating health conditions and early infant feeding behaviors, focusing on women's breastfeeding intentions and supportive hospital practices as potential mediators.

## Materials and Methods

### Conceptual Model

**Figure 1:**
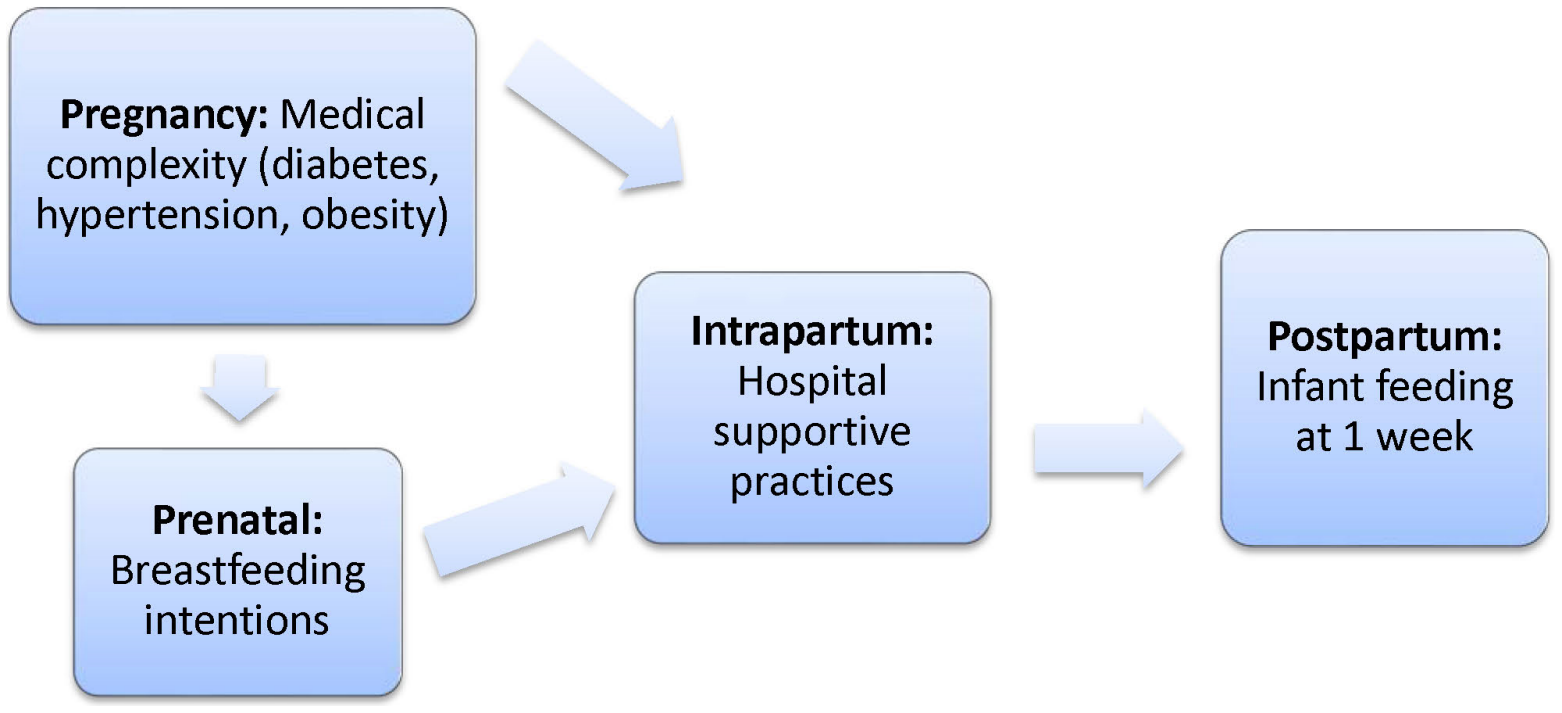
Conceptual Model.

Figure 1 presents the conceptual model for the analysis. The model focuses on women's breastfeeding intentions and hospital support practices during the intrapartum period and how these factors and their effects may differ for women who enter pregnancy with diabetes, hypertension or obesity.

### Data

Data are from the Listening to Mothers III survey, a nationally representative sample of women who gave birth to a singleton in a US hospital between July 1, 2011, and June 30, 2012 (N = 2400). The survey was commissioned by Childbirth Connection and conducted by Harris Interactive between October and December 2012. The survey documented pregnancy, labor, and birth experiences in US hospitals, including information about breastfeeding decisions and pre-existing medical conditions. Data from this survey have been widely used in clinical and public health research, including studies of breastfeeding and the role of supportive hospital practices [26], [29], [30]. However, this was the first wave of the survey to include information about medical conditions prior to pregnancy. Detailed information about the survey's methodology, implementation, and questionnaires is available at www.childbirthconnection.org/listeningtomothers/.

The data used in this analysis were de-identified. Therefore, the University of Minnesota Institutional Review Board granted this study exemption from review (Study No. 1011E92983).

### Variable Measurement

#### Pregnancy Complexity

We defined pregnancy complexity from available survey data relating to 3 common medical risk factors: (1) taking prescription medication for blood pressure during the month before pregnancy, (2) having either type 1 or type 2 diabetes before pregnancy or gestational diabetes, or (3) having a prepregnancy body mass index higher than 30. Our main analysis included a dichotomous measure of pregnancy complexity for women reporting any of these 3 conditions. We also constructed indicators for each of the conditions for separate analysis (see following description of sensitivity analyses).

#### Breastfeeding Intention

Women were asked at the time of the survey to recall their intentions about infant feeding at the end of pregnancy. We created dichotomous variables indicating (1) any intent to breastfeed (exclusively or not) and (2) women's intent to breastfeed exclusively. Supportive hospital practices and infant feeding status were assessed among women who reported any intention to breastfeed (n = 1990), and exclusive breast milk feeding status at 1 week postpartum was assessed among women who intended to exclusively breastfeed (n = 1418).

#### Supportive Hospital Practices

Among women who intended to breastfeed, we examined supportive hospital practices consistent with BFHI standards. We measured supportive hospital practices using an 8-point composite measure corresponding to 7 of the 10 BFHI steps. Measures for the remaining 3 steps were not assessed in the Listening to Mothers surveys because they require knowledge of hospital administrative policies beyond the scope of women's knowledge and experiences. However, data from these surveys have previously been used to successfully approximate BFHI hospital practices [26], [30]. See Table 1 for detailed information about the 10 BFHI steps and the 8 items assessed in the data and used in this analysis.

**Table 1:** Baby Friendly Health Initiative Composite Measure Components.

Baby Friendly Hospital Practices	Corresponding question(s) used to construct Baby Friendly Hospital Initiative Composite measure
Help mothers initiate breastfeeding within 1 hour of birth.	Baby spent 1st hour in mother's arms.
Show mothers how to breastfeed and how to maintain lactation, even if they are separated from their infants.	Hospital staff helped get started breastfeeding.
	Hospital staff showed how to position baby for breastfeeding.
Give newborn infants no food or drink other than breast milk, unless medically indicated.	Hospital staff did not provide water or formula supplements.
Practice “rooming in”—allow mothers and infants to remain together 24 hours per day.	Baby roomed with mother.
Encourage breastfeeding on demand.	Hospital staff encouraged breastfeeding on demand.
Give no pacifiers or artificial nipples to breastfeeding infants.	Hospital staff did not give baby a pacifier.
Foster the establishment of breastfeeding support groups and refer mothers to them on discharge from the hospital or clinic.	Hospital staff told about breastfeeding resources in the community.
Inform all pregnant women about the benefits and management of breastfeeding.	Not Applicable
Have a written breastfeeding policy that is routinely communicated to all health care staff.	Not Applicable
Train all health care staff in skills necessary to implement this policy.	Not Applicable

To assess general concordance with supportive breastfeeding practices in the hospital, we created a composite measure in which higher scores indicate that the woman perceived a higher level of breastfeeding-supportive hospital practices. Scores were not normally distributed, so we constructed a dichotomous variable on the basis of the top quintile of responses. Scores of 7 to 8 were categorized as “high hospital support,” indicating practices broadly consistent with BFHI standards. We also assessed the distribution of the items in the composite measure and tested the stability of the measure by modeling hospital support as a continuous variable (0–8) and by using a lower threshold (i.e., scores of 6–8 for high levels of support from the hospital). Results were robust to alternative specifications.

#### Feeding Status 1 Week Postpartum

Two dichotomous measures of infant feeding status were based on women's responses to questions regarding (1) whether they were feeding their newborn any breast milk (either exclusively or in combination with formula) 1 week postpartum, and (2) whether they were feeding their newborn breast milk only 1 week postpartum. This definition allows for both direct breastfeeding and feeding expressed breast milk to infants.

#### Control Variables

We controlled for labor and delivery factors that may affect the initiation of breastfeeding, including cesarean delivery, epidural use, and admission to a neonatal intensive-care unit [31]–[34]. We assessed these variables from maternal self-report. We also included several self-reported sociodemographic and birth-related covariates, including age; race/ethnicity (white, black, Hispanic, or other/multiple race); education (high school or less, some college, bachelor's degree, or graduate education); 4-category census region (Northeast, South, Midwest, West); nativity (foreign- or US-born); partnership status (unmarried with no partner, unmarried with partner, or married); parity (first-time pregnancy); pregnancy intention (unintended or intended pregnancy); agreement with the statement “birth is a process that should not be interfered with unless medically necessary;” doula support; and primary payer for maternity care (private, public, or out-of-pocket).

### Analysis

We first explored associations between the predictors, outcomes, and covariates for the overall sample using 1- and 2-way tabulation. We used Pearson's χ^2^ tests to determine whether differences based on pregnancy complexity were statistically significant. We used logistic regression to estimate the adjusted odds of breastfeeding intention based on pregnancy complexity. Among women intending to breastfeed, we estimated the adjusted odds of breastfeeding status 1 week postpartum. To test for mediation by hospital support, we added a variable indicating high levels of support for breastfeeding at the hospital. In the final multivariate models of breastfeeding status 1 week postpartum, we included only covariates that were statistically significantly associated with the outcomes. We conducted sensitivity analyses, estimating the same regression models using indicator variables for prepregnancy obesity, hypertension, and diabetes as the predictors rather than the combined “complex pregnancy” variable; results were substantively unchanged. All analyses used a p-value of 0.05 to determine statistical significance, were conducted using Stata v.12, and weighted to be nationally representative.

## Results

Table 2 presents the characteristics of the study population by pregnancy complexity. Overall, 36.3% of respondents had 1 or more conditions indicating a complex pregnancy (n = 871). About 8% of women were taking blood pressure medications in the month before pregnancy, 19.7% were obese, and 20.4% were diagnosed with diabetes prior to or during pregnancy. There was some overlap between conditions, particularly for diabetes and hypertension (*r* = 0.25), diabetes and obesity, (*r* = 0.09), and for hypertension and obesity (*r* = 0.04).

**Table 2:** Percentage of Women in the Study Sample (N = 2400), With a Specific Characteristic, by Pregnancy Complexity.

	Complex Pregnancy		
	No	Yes	*P* Value
Total	63.7	36.3	—
*Sociodemographic Characteristics*			
Age category			.667
18–24	31.9	31.6	
25–29	27.3	30.1	
30–34	25.7	23.1	
35+	15.0	15.2	
Race			**.023**
White	57.8	48.8	
Black	13.9	17.9	
Hispanic	22.2	24.8	
Other/multiple race	6.2	8.5	
Education			**.040**
High school or less	40.0	46.2	
Some college/associate's degree	28.9	28.0	
Bachelor's degree	18.4	16.9	
Graduate education/degree	12.8	8.9	
Region			.520
Northeast	14.5	16.4	
Midwest	23.5	21.2	
South	38.8	41.2	
West	23.2	21.2	
Foreign born	8.0	5.4	.107
Partnership status			**.003**
Unmarried with no partner	5.9	11.5	
Unmarried with partner	32.7	29.7	
Married	61.4	58.8	
*Pregnancy Characteristics*			
First-time mother	39.5	42.9	.249
Unintended pregnancy	36.1	34.1	.487
Belief that childbirth is a process that should only be interfered with if medically necessary	58.7	57.9	.797
Had doula support during labor	5.3	7.0	.281
*Health Insurance Status*			**.045**
Private	48.2	40.6	
Public	44.3	50.5	
Out-of-pocket	7.5	8.8	

Note: Percentages are weighted to be nationally representative. Bold values indicate statistically significant difference (*P*≤.05). *P* values are based on Pearson's χ^2^ tests.

Table 3 shows the distribution of breastfeeding intentions, supportive hospital practices, and infant feeding outcomes by pregnancy complexity. In bivariate associations, women with complex pregnancies were less likely to report that they intended to breastfeed (77.2% intended to do so) than women without complex pregnancies, (83.3%; *P* = .012) but there was no difference between groups in intention to exclusively breastfeed (55.7% vs. 51.0%). Overall levels of hospital breastfeeding support among women who intended to breastfeed differed by pregnancy complexity, with 14.8% of women with complex pregnancies reporting high levels of hospital support, compared with 20.4% of women without complex pregnancies (*P*  = .030). The only two statistically significant findings among the specific support measures were that women with complex pregnancies were less likely to report that their baby had spent the first hour after birth in their arms (*P* = .017) and that the hospital staff had helped them to start breastfeeding (*P* = .008). Among women planning to breastfeed, about 90% reported feeding their newborn either partially or exclusively breast milk 1 week postpartum, regardless of pregnancy complexity. Of those who intended to breastfeed exclusively, 79.5% of those without complex pregnancies and 69.4% of those with complex pregnancies were doing so (*P* = .002).

**Table 3:** Percentage of Women in the Study Population (N = 2400) With Specific Breastfeeding Behaviors, as Well as Intentions and Hospital Support, by Pregnancy Complexity.

	Complex Pregnancy		
	No	Yes	*P* Value
*Breastfeeding intentions (among all women n = 2400)*			
Intention to breastfeed, any	83.3	77.2	**.012**
Intention to breastfeed, exclusive	55.7	51	.115
*Hospital Breastfeeding Support Composite Measure (among women planning to breastfeed, n = 1990)*			
Low (0–6 steps)	79.6	85.2	
High (7–8 steps)	20.4	14.8	**.030**
*Hospital Breastfeeding Support Composite Measure Components*			
Baby in mother's arms during 1st hour after birth	51.4	43.4	**.017**
Baby roomed in with mother	63.6	59.4	.193
Hospital staff helped start breastfeeding	81.6	74.4	**.008**
Hospital staff showed how to position baby for breastfeeding	64.8	62.4	.432
Hospital encouraged breastfeeding on demand	66.4	64.6	.570
Hospital staff did NOT provide water or formula supplements	65.6	61.2	.298
Hospital staff gave information on community resources	52.2	48.7	.294
Hospital staff did NOT give baby a pacifier	58.4	62.2	.245
*Outcomes: Infant Feeding 1 Week Postpartum (among women intending to breastfeed)*			
Breastfeeding at 1 week, any (n = 1990)	91.9	89.0	.156
Breastfeeding at 1 week, exclusive (n = 1418)	79.5	69.4	**.002**

Note: Percentages are weighted to be nationally representative. Bold values indicate statistically significant difference (*P*≤.05). *P* values are based on Pearson's χ^2^ tests.

After controlling for sociodemographic and other factors (Table 4), women with more complex pregnancies were approximately 30% less likely to intend to breastfeed at all (adjusted odds ratio [AOR] = 0.71; 95% confidence interval [CI], 0.52–0.98), compared with women who had no complications entering pregnancy. However, pregnancy complexity had no independent association with intention to breastfeed exclusively.

**Table 4:** Controlled Odds of Breastfeeding Intentions by Pregnancy Complexity (N = 2400).

	*Any intention to breastfeed*	
	**AOR**	**95% CI**
Complex pregnancy	**0.71**	**(0.52–0.98)**
	*Intention to exclusively breastfeed*	
	**AOR**	**95% CI**
Complex pregnancy	0.90	(0.70**–**1.16)

Note: Models are weighted to be nationally representative. Models control for age, race/ethnicity, education, census region, nativity, partnership status, parity, unintended pregnancy, birth attitudes, and health insurance status. Bold text indicates statistically significant (*P*≤.05).

In multivariate analysis we found no relationship between complex pregnancy and whether the infant was being fed breast milk exclusively or partially 1 week postpartum (Table 5) after controlling for the same sociodemographic and clinical covariates. In subsequent models, we also controlled for supportive hospital practices to examine potential mediation. Babies whose mothers received high levels of hospital support for breastfeeding were 4 times more likely to receive at least some breast milk 1 week postpartum. Among women who intended to exclusively breastfeed, those with complex pregnancies had more than 30% lower odds of feeding their infants breast milk only (AOR = 0.68; 95% CI, 0.47–0.98). High levels of hospital support for breastfeeding were associated with nearly 3 times the odds of exclusive breastfeeding 1 week postpartum (AOR = 2.79; 95% CI, 1.77–4.39). When these factors were included simultaneously, the association between pregnancy complexity and lower odds of exclusive breastfeeding remained similar (AOR = 0.69; 95% CI, 0.48–1.00).

**Table 5:** Controlled Odds of Infant Feeding Status at 1 Week by Pregnancy Complexity and Supportive Hospital Practices.

	*Any Breastfeeding 1 Week Postpartum (n = 1990)*			
	AOR	95% CI	AOR	95% CI
				
Complex pregnancy	0.81	(0.49**–**1.34)	0.82	(0.50**–**1.36)
High supportive hospital practices			**4.03**	**(1.81–8.94)**
	*Exclusive Breastfeeding 1 Week Postpartum (n = 1418)*			
	AOR	95% CI	AOR	95% CI
Complex pregnancy	**0.68**	**(0.47–0.98)**	**0.69**	**(0.48–1.00)**
High supportive hospital practices			**2.68**	**(1.70–4.23)**

Note: Models are weighted to be nationally representative. Models control for age, race/ethnicity, education, census region, nativity, partnership status, parity, unintended pregnancy, birth attitudes, health insurance status, cesarean delivery and doula support. Bold text indicates statistically significant (*P*≤.05).

## Discussion

The study examined the effect of entering pregnancy with medical complications on infant feeding practices among those who intended to breastfeed either at all or exclusively, and the influence of hospital practices on those decisions. Women with hypertension or diabetes or those who were obese when they became pregnant were less likely to intend to breastfeed than women whose pregnancies were not complicated by these conditions. Our results also show that women with complex pregnancies who planned to exclusively breastfeed were substantially less likely to do so 1 week postpartum than women without pregnancy complications, even after accounting for supportive hospital practices.

The findings point to clear opportunities for intervention and support during pregnancy and immediately after giving birth. Obstetricians, midwives, family physicians, and pediatricians should be aware that women with complex pregnancies are less likely to plan to breastfeed and are less likely to receive recommended hospital-based support.

Multiple research studies and systematic reviews confirm that simply counseling women to breastfeed is not sufficient for encouraging women to breastfeed; rather, tailored support offered both prenatally and postpartum is most effective in supporting pregnant women to set and attain breastfeeding goals [35]–[37]. Clinicians should discuss breastfeeding intentions when establishing relationships with patients prenatally, including consultation on plans for the use of anti-diabetic or anti-hypertensive medications compatible with a mother's intentions, and follow up to ensure that women with complicated pregnancies have access to breastfeeding support in the hospital [38]. It is also important to address breastfeeding intentions and provide encouragement and support at the time of delivery, given that delivery third of US women lack a prior relationship with the clinician attending their delivery [39]. Providing encouragement and support at the time of delivery may be particularly important for women with complex pregnancies who may be transferred to higher acuity care teams at delivery [40]–[42]. The results of our analysis suggest that women who are nonwhite, less educated, unmarried with no partner, and using public health insurance are more likely to be obese or to develop hypertension or diabetes prior to pregnancy, so it may be helpful to target outreach and support efforts to these groups.

Our findings are consistent with prior research showing that BFHI-consistent hospital practices help to promote early breastfeeding success [24]–[27]. Women who reported a high number of BFHI-consistent hospital practices were 3 times more likely to exclusively breastfeed than were those who reported a lower number of BFHI-consistent practices. Women who entered pregnancy with hypertension, diabetes, or obesity were significantly less likely to report experiencing the BFHI-consistent hospital practices of having their baby in their arms during the first hour after birth and having hospital staff help them start breastfeeding. Therefore, hospitals and clinicians alike should pay particular attention to showing women with complex pregnancies how to breastfeed (including expressing breast milk for bottle or syringe feeding [43]) and supporting early breastfeeding efforts, including after cesarean delivery [44], [45].

Breastfeeding support should be incorporated into clinical and hospital policies, with emphasis on women with complex pregnancies [46]. Postpartum care management or obstetric/neonatal discharge guidelines for obese women and those with diabetes or hypertension could explicitly include discussions of breastfeeding and information about community-based resources. In addition, compliance with BFHI steps should be promoted in more hospitals, consistent with the federal Healthy People 2020 goals, as should practices that have been shown to improve breastfeeding outcomes despite not being part of the BFHI scale, such as skin-to-contact between women and their infants immediately after birth [47], [48]. Hospital should also be aware of well-intentioned practices to support breastfeeding that women may in fact experience negatively. Hands-on-breast approaches to breastfeeding support, for instance, may be considered unpleasant and disrespectful by some women [49]. Hospitals and staff should continue to maintain open communication with women about the best ways to support their breastfeeding intentions.

### Limitations

Although providing a rich source of data on breastfeeding from a patient perspective, the Listening to Mothers surveys have certain limitations that warrant discussion. These data are based on retrospective self-reports, leaving room for potential recall bias and social desirability bias. Although the survey contained some information about health conditions, assessment of these conditions is based on maternal self-report. In addition to the complications we included in our analysis, other maternal, fetal, and neonatal medical conditions or complications that arise during labor and delivery could also be associated with breastfeeding intention and practices. Finally, our construction of the BFHI composite measure relied on maternal perception of proxies for 7 of the 10 BFHI steps. However, several of the 10 BFHI steps include questions about hospital policy, of which many women may not be aware.

## Conclusion

Breastfeeding is beneficial for women and infants, and medical contraindications are rare. Complications that occur during pregnancy, labor, and delivery may hinder breastfeeding, but supportive hospital practices may facilitate breastfeeding for women who intend to breastfeed.

We distinguished breastfeeding intentions and early feeding patterns for women with complex pregnancies and found lower odds of intending to breastfeed and decreased chances of early exclusive breastfeeding, even after accounting for supportive hospital practices, which were associated with greater breastfeeding success. Therefore, it is important to support women with medically complex pregnancies in overcoming potential challenges to breastfeeding.
